# Balanced inpatient and outpatient reimbursement versus hospitalization-favored insurance reduces Crohn disease costs and improves biologic drug persistence: A mediation analysis

**DOI:** 10.1097/MD.0000000000044475

**Published:** 2025-09-12

**Authors:** Jihao Shi, Yipeng Pan, Yue Gao, Xiaohan Hu, Yongjia Zhuo, Kangchen Lv, Qian Cao

**Affiliations:** a Inflammatory Bowel Disease Center, Sir Run Run Shaw Hospital, Zhejiang University School of Medicine, Hangzhou, Zhejiang, China; b Department of Gastroenterology, Sir Run Run Shaw Hospital, Zhejiang University School of Medicine, Hangzhou, Zhejiang, China; c Health Economic Research Institute, School of Pharmaceutical Sciences, Sun Yat-Sen University, Guangzhou, Guangdong, China.

**Keywords:** Crohn disease, health insurance, treatment cost

## Abstract

Crohn disease (CD) treatment incurs high costs due to biologics and frequent hospitalizations. Insurance policies favoring hospitalization may increase costs by discouraging outpatient care. We conducted a mediation analysis to examine whether balanced inpatient and outpatient reimbursement reduces CD treatment costs and improves biologic persistence, mediated by outpatient treatment. In this retrospective cohort study, we analyzed 133 CD patients initiating ustekinumab (UST) in 2022 at a tertiary hospital. The exposure was balanced insurance coverage (Hangzhou Medical Insurance) versus hospitalization-favored coverage (Zhejiang Province Card Insurance). The mediator was outpatient UST treatment. Primary outcome was total CD-related treatment costs; secondary outcomes included CD-related inpatient stays, outpatient visits, and UST persistence. Causal mediation analysis, adjusted for age, gender, and disease severity, estimated natural direct effects, natural indirect effects (NIE), and proportion mediated. Patients with balanced coverage had lower total CD-related costs (adjusted mean difference: −CNY 6362; 95% CI: −11,658 to − 1067), fully mediated by increased outpatient treatment (NIE: −CNY 10,125; 95% CI: −15,920 to − 4330; proportion mediated: 100%). They had fewer inpatient stays (−1.64; 95% CI: −2.10 to − 1.18; NIE: −0.84; 95% CI: −1.33 to − 0.37; proportion mediated: 50%) and more outpatient visits (6.53; 95% CI: 5.48 to 7.57). Balanced coverage reduced UST discontinuation risk by 73% (adjusted HR: 0.27; 95% CI: 0.10–0.74), not mediated by outpatient treatment. Balanced insurance coverage reduces CD treatment costs and inpatient stays via increased outpatient treatment and improves UST persistence independently. Policymakers should equalize coverage to optimize CD care efficiency.

## 1. Introduction

Inflammatory bowel disease (IBD), including Crohn disease (CD), is a chronic condition causing digestive tract inflammation, resulting in severe symptoms, increased mortality, and frequent healthcare use.^[1,2]^ In China, the prevalence of CD has increased rapidly over recent decades, with an estimated 30,000 to 50,000 patients affected nationwide, driven by urbanization and improved diagnostic capabilities.^[3,4]^

Biologic drugs, such as tumor necrosis factor inhibitors, effectively reduce symptoms and the need for surgery by targeting inflammation.^[5,6]^ However, these treatments are expensive, driving up healthcare costs globally.^[7]^ Biologics can be administered via injections under the skin (subcutaneous, SC) or intravenous (IV) infusions. SC injections are as effective and safe as IV infusions, better tolerated, and reduce healthcare costs by enabling treatment in outpatient visits rather than hospitalizations.^[8]^ For patients, SC therapy offers convenience, eliminating travel to infusion centers and minimizing disruptions to work or education.^[9]^ In China, however, most CD patients receive ongoing biologic treatment during hospitalization due to insurance policies favoring hospitalization care. For example, in Zhejiang province, insurance covers 83% of hospitalization costs but only 65% of outpatient costs, increasing patients’ out-of-pocket expenses for outpatient visits.^[10,11]^ This disparity may lead patients to prefer hospitalization stays, increasing costs and reducing convenience.

Since 2021, China has expanded insurance coverage for outpatient treatment of chronic diseases like CD to reduce hospitalization reliance and improve access.^[12]^ However, these policies vary by region and often exclude treatment outside patients’ home cities.^[12]^ In the US, studies have shown that health insurance increases healthcare utilization and improves outcomes,^[13–15]^ but findings are inconsistent, with some contradicting the RAND Health Insurance Experiment. For pediatric CD, publicly insured children have greater access to biologics but higher hospitalization and emergency visit rates, suggesting that insurance type influences utilization patterns, though the role of specific policy features remains unclear. These studies highlight controversies regarding the causal impact of insurance design on costs and outcomes, particularly in non-US contexts and for treatment persistence.^[13,16]^ We therefore hypothesized that unequal coverage for hospitalization versus outpatient care increases CD treatment costs by favoring inpatient SC biologic injections. Using Ustekinumab (UST), an SC biologic, as an example, we examined whether an insurance policy with balanced inpatient and outpatient reimbursement reduces total CD treatment costs and improves treatment persistence, and whether these effects are explained by a shift to outpatient treatment. Mediation analysis was employed to quantify the extent to which outpatient treatment explains cost and persistence outcomes, providing insights into the causal pathways of insurance policy effects.^[17]^

## 2. Methods

This retrospective cohort study, approved by the Ethics Committee of Sir Run Run Shaw Hospital, adhered to the Declaration of Helsinki and followed the AGReMA Statement for reporting mediation analyses.^[17]^ We conducted a natural experiment using causal mediation analysis (CMA) to assess whether a shift to outpatient treatment mediated the effect of insurance type on CD treatment costs and persistence

### 2.1. Data source and study population

We used data from the IBD registry at Zhejiang University-affiliated Sir Run Run Shaw Hospital’s tertiary IBD Center, established in 1999. The registry includes 7953 IBD patients (4919 with CD and 3034 with ulcerative colitis [UC]), representing approximately 4% to 9% of the estimated 94,120–192,380 IBD patients in China, reflecting the center’s role as a major referral hub.^[1]^ It documents over 180,000 hospitalization and outpatient visits and an average follow-up of 12.3 years, with data up to 2022 for this study. It collects longitudinal data on diagnosis date, disease characteristics (severity, location), medication use (e.g., biologics), surgeries, and recent lab or endoscopy results, extracted from the hospital’s information system.^[18]^

We included CD patients who: were diagnosed with CD, were continuously enrolled in one of 2 public insurance plans (Hangzhou Medical Insurance or Zhejiang Province Card) during 2022, and initiated UST, an injectable biologic, for ongoing treatment in 2022.

### 2.2. Exposure and comparator

The exposure was Hangzhou Medical Insurance, which offers similar coverage for CD treatment in outpatient (76%–92%, depending on hospital level and employment status, no cap) and inpatient settings (82%–92%, capped at CNY 400,000 annually).^[19]^ The comparator was Zhejiang Province Card Insurance, which covers inpatient CD treatment but not outpatient care. There was no specific requirement for the duration of enrollment in either insurance plan at the time of enrollment (UST initiation); patients were assumed to be continuously enrolled in one of these plans throughout 2022 to ensure consistent insurance status during the study period. Both groups were similar in age, gender, and disease characteristics at baseline (Table 1). For cost outcomes, patients were followed from the start of UST treatment, defined as the index date (the date of UST initiation) to December 31, 2022, including those who discontinued UST, to capture the full impact of treatment patterns, including early discontinuation, on CD-related healthcare costs.

**Table 1:** Baseline characteristics of Crohn disease patients initiating ustekinumab by insurance type at Sir Run Run Shaw Hospital, Hangzhou, China, 2022.

Baseline characteristic	Participants, No. (%)		
	Exposed group (n = 65)	Control group (n = 68)	
Age of patients, mean (SD), year	35.61 (1.68)	34.63 (1.54)	0.6658
Age at first diagnosis of CD			
≤16	6 (9.23)	5 (7.35)	0.926
17–40	45 (69.23)	48 (70.59)	
>40	14 (21.54)	15 (22.06)	
Sex			
Female	14 (21.54)	24 (35.29)	0.079
Male	51 (78.46)	44 (64.71)	
Follow-up, mean (SD), days	247.95 (79.51)	250.60 (83.54)	0.8518
Location*			
L1	19 (29.23)	22 (32.35)	0.618
L1 + L4†	9 (13.85)	7 (10.29)	
L2	0	2 (2.94)	
L3	24 (36.92)	26 (38.24)	
L3 + L4†	13 (20)	11 (16.18)	
Behaviour‡			
B1	9 (13.85)	7 (10.29)	0.352
B1p§	16 (24.62)	19 (27.94)	
B2	10 (15.38)	11 (16.18)	
B2B3	1 (1.54)	4 (5.88)	
B2B3p§	4 (6.15)	10 (14.71)	
B2p§	18 (27.69)	13 (19.12)	
B3	2 (3.08)	0	
B3p§	5 (7.69)	4 (5.88)	
Disease activity			
Moderate	23 (35.38)	27 (39.71)	0.251
Remission	2 (3.08)	8 (11.76)	
Mild	23 (35.38)	19 (27.94)	
Severe	1 (1.54)	0 (0)	
NA∥	16 (24.62)	14 (20.59)	
Gastrointestinal surgery history	25 (38.46)	36 (52.94)	0.094
Perianal surgery history	24 (36.92)	28 (41.18)	0.615
Number of biologics used			
0	33 (50.77)	31 (45.59)	0.362
1	23 (35.38)	23 (33.82)	
2	9 (13.85)	11 (16.18)	
3	0	3 (4.41)	

* L1 ileal, L2 colonic, L3 ileocolonic, L4 isolated upper disease.^23^

† L4 is a modifier that can be added to L1–L3 when concomitant upper gastrointestinal disease is present.^23^

‡ B1 non‐structuring, non‐penetrating, B2 structuring, B3 penetrating, p perianal disease modifier.^23^

§ “p” is added to B1–B3 when concomitant perianal disease is present.^23^

∥ NA: not available.

### 2.3. Outcomes

The primary outcome was total CD-related treatment costs, defined as the sum of hospitalization, outpatient, and emergency room costs from the index date (UST initiation) to December 31, 2022. Secondary outcomes included CD-related costs by setting, number and duration of inpatient stays, number of outpatient visits, and UST persistence, defined as time to discontinuation during follow-up. Treatment persistence was selected as a key outcome because sustained biologic therapy is critical for CD control, reducing complications and healthcare costs, yet it may be influenced by economic barriers such as insurance coverage. Prior studies indicate that high out-of-pocket costs due to limited insurance coverage can lead to treatment discontinuation or switching to less expensive regimens in chronic diseases, including IBD.^[1,16]^ In pediatric CD, publicly insured patients had greater biologic access but higher hospitalization rates, suggesting cost-driven shifts in treatment settings.^[13]^ We hypothesized that balanced reimbursement would enhance UST persistence by facilitating outpatient care, reducing cost barriers to sustained therapy.

#### 2.3.1. Confounders and mediator

As insurance types are determined by patients’ city of residence, self-selection bias was minimal. However, patients from outside Hangzhou (control group) might have more severe CD due to seeking care at a tertiary center outside their living city. We compared baseline disease severity, surgery history, and prior biologic use between groups. Socioeconomic differences between Hangzhou and Zhejiang province were not expected to substantially confound outcomes, despite Hangzhou having a higher per capita GDP in 2022 (CNY 152,588 vs CNY 118,800 for Zhejiang province). Both regions are relatively affluent, and the study design minimized potential socioeconomic confounding by comparing similar patient groups. Additional confounders included age, gender, CD onset age, disease location and behavior, and surgical history.

The mediator was the use of outpatient care for all UST treatments, defined as receiving all UST doses in outpatient settings (1) versus at least 1 dose in inpatient settings (0) during the follow-up period.

### 2.4. Statistical methods

Baseline characteristics and cost differences were compared using Student *t* test (for continuous variables with equal variance), Kruskal-Wallis test (unequal variance), or chi-square test (categorical variables). Treatment persistence was visualized using Kaplan–Meier curves stratified by insurance type. Cox proportional hazards regression, adjusted for imbalanced baseline covariates, estimated HRs for UST discontinuation. Baseline gender and gastrointestinal surgery history were selected as key confounders for adjustment in all analyses due to their potential to influence healthcare utilization and treatment persistence in CD, based on observed differences in baseline characteristics (Table 1).^[13,14]^ For the persistence outcome (time to UST discontinuation), patients were censored at the earliest occurrence of UST discontinuation, treatment switch, loss to follow-up, or the study end date (December 31, 2022). Adjusted absolute differences quantified excess costs or healthcare use associated with the exposure. All tests were 2-sided, with significance at *P* < .05, performed using Stata 13 (StataCorp, College Station, TX, USA).

### 2.5. Mediation analysis

We conducted CMA to evaluate whether a shift to outpatient CD treatment mediated the effect of insurance type on CD-related treatment costs and other outcomes.^[20]^ This approach decomposes the total effect (TE) of insurance type on outcomes into a natural direct effect (NDE), representing the effect of insurance type not mediated by outpatient treatment, and a natural indirect effect (NIE), capturing the effect mediated through outpatient treatment. The analysis is grounded in a counterfactual framework, where counterfactual outcomes represent the potential outcomes under different levels of exposure (insurance type) and mediator (outpatient treatment). For instance, (Y^w,m^) denotes the counterfactual outcome for an outcome (Y) under insurance type (w) and outpatient treatment status (m), and (M^w^) represents the counterfactual value of the mediator under insurance type (w).

The NDE is defined as (Y ^w=1, Mw=0^—Y ^w=0, M w=0^), comparing outcomes when the mediator is fixed at the level it would take under the control condition (no insurance or reference insurance type) while varying the exposure. The NIE is defined as (Y ^w=1, Mw=1^—Y ^w=1, Mw=0^), capturing the effect of changing the mediator while fixing the exposure. The TE is the sum of NDE and NIE: (TE = Y ^w=1^—Y ^w=0^ = NDE + NIE). A visual representation of the mediation model is provided in Figure S1, Supplemental Digital Content, https://links.lww.com/MD/P949 illustrating the pathways from insurance type to outcomes via outpatient treatment. We controlled for potential confounders, including age, gender, and socioeconomic status, to ensure accurate inference. Proportion mediated, which is the proportion of the TE explained by outpatient treatment, was calculated as the ratio of the NIE to the TE, expressed as a percentage.

The mediation analysis assumed no unmeasured confounding between exposure and outcomes, exposure and mediator, and mediator and outcomes, conditional on measured confounders (age, gender, CD onset age, disease location, behavior, surgical history).

### 2.6. Additional analyses

We conducted additional analyses to assess the robustness of findings: a restricted analysis limited to patients receiving all UST treatment in a single setting (either inpatient or outpatient, excluding those with mixed inpatient and outpatient treatments) to evaluate the impact of consistent treatment settings on costs, resource use, and mediation effects (Tables S1 and 3, Supplemental Digital Content, https://links.lww.com/MD/P950), and an adjusted analysis controlling for baseline gender and gastrointestinal surgery history to account for potential confounding (Table S2, Supplemental Digital Content, https://links.lww.com/MD/P950).

## 3. Results

### 3.1. Participant selection

**Figure 1.**
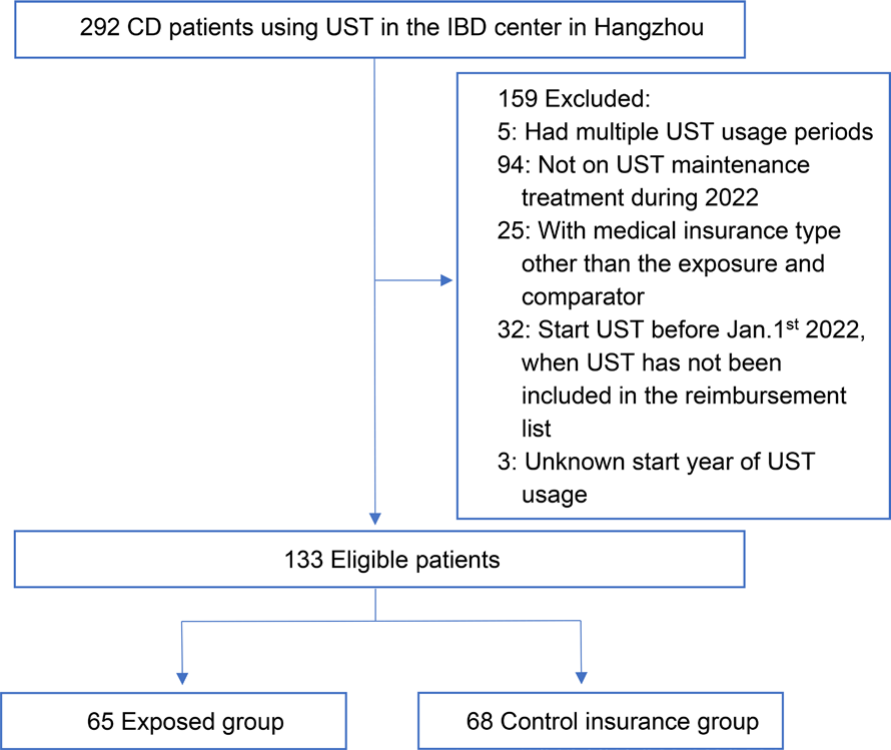
Flow diagram of cohort creation for Crohn disease patients initiating ustekinumab at Sir Run Run Shaw Hospital, Hangzhou, China, 2022.

We included 133 CD patients who met the inclusion criteria: diagnosed with CD, continuously enrolled in either Hangzhou Medical Insurance (exposed group, n = 65, 48.9%) or Zhejiang Province Card Insurance (control group, n = 68, 51.1%), and initiated UST in 2022 at the IBD Center of Sir Run Run Shaw Hospital. The cohort creation process, including patient selection and exclusion criteria, is detailed in Figure 1. Patients were followed from the UST initiation date (index date) to December 31, 2022, with a median follow-up of 255 days (IQR: 171–321, approximately 8.5 months).

### 3.2. Participant characteristics

Baseline characteristics of the 133 included patients, stratified by insurance type, are presented in Table 1. The exposed and control groups were similar in age (mean: 35.61 years [SD: 1.68] vs 34.63 years [SD: 1.54]), age at CD diagnosis (≤16 years: 9.23% vs 7.35%; 17–40 years: 69.23% vs 70.59%; >40 years: 21.54% vs 22.06%), and follow-up duration (mean: 247.95 days [SD: 79.51] vs 250.60 days [SD: 83.54]). Sex distribution showed a slightly higher proportion of males in the exposed group (78.46% vs 64.71%), though this difference was not statistically significant. Disease location (Montreal classification: L1 [ileal]: 29.23% vs 32.35%; L3 [ileocolonic]: 36.92% vs 38.24%; L1 + L4 or L3 + L4: 33.85% vs 26.47%) and behavior (B1 [non-structuring, non-penetrating]: 13.85% vs 10.29%; B2 [structuring]: 15.38% vs 16.18%; B3 [penetrating]: 3.08% vs 0%) were comparable, with minor differences in perianal disease modifiers. Disease activity at baseline was balanced (moderate: 35.38% vs 39.71%; mild: 35.38% vs 27.94%; remission: 3.08% vs 11.76%; severe: 1.54% vs 0%), with some missing data (24.62% vs 20.59%). Gastrointestinal surgery history (38.46% vs 52.94%) and perianal surgery history (36.92% vs 41.18%) were slightly more frequent in the control group but not significantly different. Prior biologic use was similar (no biologics: 50.77% vs 45.59%; 1 biologic: 35.38% vs 33.82%; 2 or more biologics: 13.85% vs 20.59%). These findings indicate that key confounders, including age, disease characteristics, and treatment history, were well-balanced between groups, minimizing potential selection bias.

### 3.3. Healthcare expenditure and resource use outcomes

The exposed group (Hangzhou Medical Insurance) incurred lower CD-related hospitalization costs compared to the control group (Zhejiang Province Card Insurance) (CNY 10,114.74 [95% CI: 6813.54–13,415.94] vs CNY 16,129.78 [95% CI: 12,115.69–20,143.88]; mean difference: −CNY 6015.04 [95% CI: −11,189.46 to − 840.62]; adjusted mean difference: −CNY 6601.77 [95% CI: −11,902.64 to − 1300.90]) and higher CD-related outpatient costs (CNY 460.91 [95% CI: 322.76–599.06] vs CNY 240.75 [95% CI: 123.03–358.47]; mean difference: CNY 220.16 [95% CI: 40.92–399.40]; adjusted mean difference: CNY 226.64 [95% CI: 42.29–411.00]). Emergency room costs were similar (CNY 64.89 [95% CI: 18.28–111.49] vs CNY 60.17 [95% CI: −0.22–120.55]; mean difference: CNY 4.72 [95% CI: −71.33 to 80.77]; adjusted mean difference: CNY 12.94 [95% CI: −65.19 to 91.06]). For resource use, the exposed group had fewer CD-related inpatient stays (2.57 [95% CI: 2.34–2.80] vs 4.26 [95% CI: 3.89–4.64]; mean difference: −1.70 [95% CI: −2.14 to − 1.25]; adjusted mean difference: −1.64 [95% CI: −2.10 to − 1.18]), shorter inpatient length of stay (5.28 days [95% CI: 4.22–6.34] vs 7.53 days [95% CI: 6.46–8.60]; mean difference: −2.25 [95% CI: −3.74 to − 0.76]; adjusted mean difference: −2.02 [95% CI: −3.56 to − 0.50]), and more CD-related outpatient visits (8.58 [95% CI: 7.65–9.52] vs 2.28 [95% CI: 1.80–2.76]; mean difference: 6.31 [95% CI: 5.25–7.36]; adjusted mean difference: 6.53 [95% CI: 5.48–7.57]). Emergency room visit numbers were comparable (0.23 [95% CI: 0.08–0.38] vs 0.21 [95% CI: 0.04–0.37]; mean difference: 0.02 [95% CI: −0.20 to 0.25]; adjusted mean difference: 0.04 [95% CI: −0.19 to 0.28]). Adjusted analyses, accounting for baseline gender and gastrointestinal surgery history, confirmed the robustness of these findings (Table 2). The restricted analysis, limited to patients receiving all UST treatment in 1 setting (inpatient or outpatient, n = not specified), showed consistent results (Table S1, Supplemental Digital Content, https://links.lww.com/MD/P950), with lower hospitalization costs (mean difference: −CNY 5994.05; 95% CI: −11,133.65 to − 854.46), higher outpatient costs (mean difference: CNY 221.55; 95% CI: 30.16–412.95), and fewer inpatient stays (mean difference: −1.69; 95% CI: −2.15 to − 1.23) in the exposed group, reinforcing the main findings.

**Table 2:** Healthcare expenditure and resource use outcomes for Crohn disease patients initiating ustekinumab by insurance type at Sir Run Run Shaw Hospital, Hangzhou, China, 2022.

	Cost and HRU, mean (95% CI)				
	Total included patients	Balanced insurance group (n = 65)	Inpatient-only group (n = 68)	Cost/HRU difference	Adjusted cost/HRU difference†
Healthcare expenditure outcome, CNY					
Total CD treatment cost	13,600.92	10,640.53	16,430.70	−5790.17	−6362.20
95% CI*	(10,979.24–16,222.60)	(7366.32–13,914.74)	(12,403.20–20,458.20)	(−10,959.22 to −621.11)‡	(−11,657.77 to −1066.61)‡
CD-related hospitalization cost	13,190.10	10,114.74	16,129.78	−6015.04	−6601.77
95% CI*	(10,562.06–15,818.15)	(6813.54–13,415.94)	(12,115.69–20,143.88)	(−11,189.46 to −840.62)‡	(−11,902.64 to −1300.90)‡
CD-related outpatient cost	348.35	460.91	240.75	220.16	226.64
95% CI*	(257.11–439.59)	(322.76–599.06)	(123.03–358.47)	(40.92–399.40)^c^	(42.29–411.00)‡
CD-related emergency room cost	62.47	64.89	60.17	4.72	12.94
95% CI*	(24.60–100.34)	(18.28–111.49)	(−.22 to 120.55)	(−71.33 to 80.77)	(−65.19 to 91.06)
Healthcare resource use outcome					
Number of inpatient stays	3.44	2.57	4.26	−1.70	−1.64
95% CI*	(3.17, 3.70)	(2.34–2.80)	(3.89–4.64)	(−2.14 to −1.25)‡	(–2.10 to 1.18)‡
CD-related outpatient visit number	5.36	8.58	2.28 (1.80–2.76)	6.31 (5.25–7.36)‡	6.53 (5.48–7.57)‡
95% CI*	(4.61, 6.11)	(7.65, 9.52)	–	–	–
CD-related emergency room visit number	0.22	0.23	0.21	0.02	0.04
95% CI*	(0.11–0.33)	(0.08–0.38)	(0.04–0.37)	(−0.20 to 0.25)	(−0.19 to 0.28)
CD-related inpatient length of stay	6.43	5.28	7.53	−2.25	−2.02
95% CI*	(5.66–7.20)	(4.22–6.34)	(6.46–8.60)	(−3.74 to −0.76)^c^	(−3.56 to −0.50)‡

CI = confidence interval, CNY = Chinese Yuan, HRU = health resource use.

* Numbers in brackets represent 95% confidence intervals unless otherwise specified.

† Adjusted for baseline gender and gastrointestinal surgery history. The balanced insurance group refers to Hangzhou Medical Insurance (similar coverage for inpatient and outpatient care); the inpatient-only group refers to Zhejiang Province Card Insurance (covers inpatient care but not outpatient care).

‡ Statistically significant difference at *P* < .05.

### 3.4. Treatment persistence

**Figure 2.**
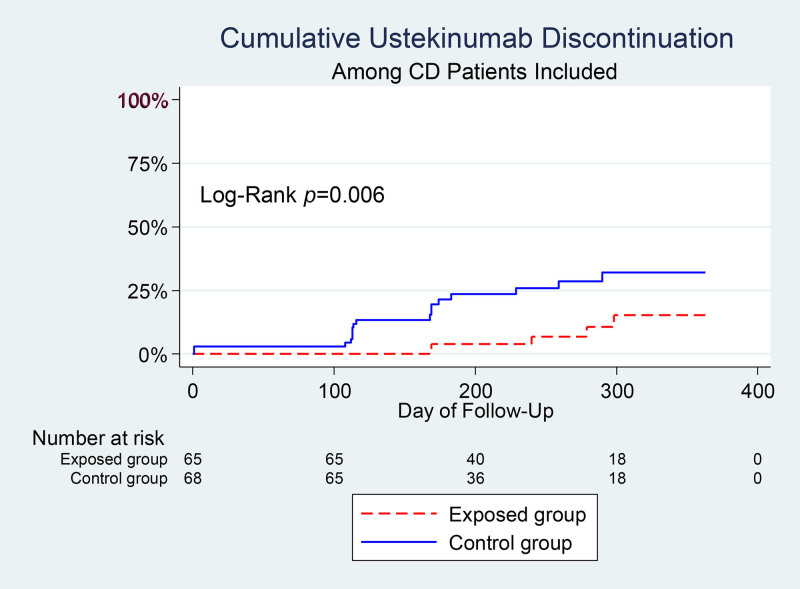
Kaplan–Meier curves for ustekinumab persistence in Crohn disease.

The exposed group had a 72.8% lower risk of UST discontinuation compared to the control group (7.7% vs 25%; adjusted HR: 0.27 [95% CI: 0.10–0.74]), as visualized in Kaplan–Meier curves (Fig. 2).

#### 3.4.1. Mediation analysis

CMA assessed whether a shift to outpatient UST treatment mediated the effect of insurance type (Hangzhou Medical Insurance vs Zhejiang Province Card Insurance) on healthcare expenditure, resource use, and treatment persistence outcomes. Results are presented as NDE, NIE via outpatient treatment, and proportion mediated where applicable (Table 3). Unadjusted results are reported in Table 3, with adjusted results (for baseline gender and gastrointestinal surgery history) in Table S2, Supplemental Digital Content, https://links.lww.com/MD/P950 and restricted analysis results (for patients receiving all UST treatment in 1 setting) in Table S3, Supplemental Digital Content, https://links.lww.com/MD/P950. A visual representation of the mediation model is provided in Figure S1, Supplemental Digital Content, https://links.lww.com/MD/P949. For outcomes where NDE and NIE operated in opposite directions, the proportion mediated was not calculated, as it can be misleading, consistent with AGReMA guidance.^[16]^

**Table 3:** Mediation analysis of outpatient treatment effects on outcomes for Crohn disease patients initiating ustekinumab by insurance type at Sir Run Run Shaw Hospital, Hangzhou, China, 2022.

Treatment setting’s mediating effect on different outcomes*†	Mean difference (95% CI)/(HR, 95% CI)			Proportion mediated (%)‡
	Total effect	Natural direct effect	Natural indirect effect	
Healthcare expenditure outcome, CNY				
CD-related treatment cost	−5790.17 (−10,954.84 to −625.49)§	4334.92 (−3062.95 to 11,732.80)	−10,125.09 (−15,920.24 to −4329.94)§	NA
CD-related hospitalization cost	−6015.04 (−11,185.20 to −844.88)§	4313.07 (−3079.96 to 11,706.09)	−10,328.11 (−16,129.53 to −4526.68)§	NA
CD-related outpatient cost	220.16 (41.17–399.14)§	52.44 (−215.13 to 320.02)	167.71 (−34.36 to 369.79)	76.18
CD-related emergency room cost	4.72 (−71.21 to 80.65)	−30.59 (−144.89 to 83.71)	35.31 (−50.39 to 121.00)	NA
Healthcare resource use outcome				
CD-related hospitalization number	−1.70 (−2.14 to −1.25)	−0.85 (−1.46 to −0.23)^d^	−0.85 (−1.33 to −0.37)^d^	49.99§
CD-related outpatient visit number	6.31 (5.25–7.36)§	5.26 (3.69–6.83)§	1.05 (−0.14 to 2.23)∥	16.61¶
CD-related emergency room visit number	0.02 (−0.20 to 0.25)	−0.14 (−0.47 to 0.20)	0.16 (−0.09 to 0.42)	NA
CD-related inpatient length of stay	−2.25 (−3.74 to −0.76)§	−1.07 (−3.30 to 1.15)	−1.18 (–2.85 to 0.50)	52.28
Persistence (HR)	0.48 (0.36–0.63)§	0.37 (0.366–0.38)§	1.28 (0.99–1.66)#	NA

CD =Crohn disease, CI = confidence interval, CNY = Chinese Yuan.

* The exposed group is compared with the control group.

† The mediating effect of receiving all treatment under outpatient setting during follow-up is compared with receiving at least 1 treatment under inpatient setting (reference group).

‡ Proportion mediated: the measure is problematic when the natural direct effect and natural indirect effect operate in different directions. One can then obtain a proportion mediated much larger than 100%, and the measure is no longer really meaningful.

§ Statistically significant effect at a *P* value of <.05.

∥ *P* value: .084.

¶ *P* value: .09.

# *P* value: .064.

For CD-related treatment costs, the unadjusted TE of balanced insurance was a reduction of − CNY 5790.17 (95% CI: −10,954.84 to − 625.49), with an NIE of − CNY 10,125.09 (95% CI: −15,920.24 to − 4329.94) and a nonsignificant NDE (CNY 4334.92; 95% CI: −3062.95 to 11,732.80), indicating full mediation by outpatient treatment. Adjusted analysis (Table S2, Supplemental Digital Content, https://links.lww.com/MD/P950) showed a similar NIE (−CNY 10,219.22; 95% CI: −16,043.37 to − 4395.08), and the restricted analysis (Table S3, Supplemental Digital Content, https://links.lww.com/MD/P950) strengthened the NIE (−CNY 16,575.26; 95% CI: −24,500.37 to − 8650.15), confirming that the cost reduction was driven by increased outpatient treatment reducing reliance on inpatient care.

For CD-related hospitalization costs, the unadjusted TE was − CNY 6015.04 (95% CI: −11,185.20 to − 844.88), with an NIE of − CNY 10,328.11 (95% CI: −16,129.53 to − 4526.68) and a nonsignificant NDE (CNY 4313.07; 95% CI: −3079.96 to 11,706.09), indicating full mediation. Adjusted results (Table S2, Supplemental Digital Content, https://links.lww.com/MD/P950) were consistent (NIE: −CNY 10,425.14; 95% CI: −16,255.61 to − 4594.67), and the restricted analysis (Table S3, Supplemental Digital Content, https://links.lww.com/MD/P950) showed a stronger NIE (−CNY 16,888.80; 95% CI: −24,812.81 to − 8964.79), reinforcing that outpatient treatment reduced hospitalization costs.

For CD-related outpatient costs, the unadjusted TE was an increase of CNY 220.16 (95% CI: 41.17 to 399.14), with an NIE of CNY 167.71 (95% CI: −34.36 to 369.79; *P* = .104) and a nonsignificant NDE (CNY 52.44; 95% CI: −215.13 to 320.02), with a proportion mediated of 76.18%. Adjusted analysis (Table S2, Supplemental Digital Content, https://links.lww.com/MD/P950) showed a similar NIE (CNY 169.29; 95% CI: −34.15 to 372.73), and the restricted analysis (Table S3, Supplemental Digital Content, https://links.lww.com/MD/P950) showed a significant NIE (CNY 292.39; 95% CI: 30.15 to 554.63; *P* = .029), indicating that increased outpatient visits drove higher outpatient costs.

For CD-related emergency room costs, the unadjusted TE was not significant (CNY 4.72; 95% CI: −71.21 to 80.65), with nonsignificant NDE (−CNY 30.59; 95% CI: −144.89 to 83.71) and NIE (CNY 35.31; 95% CI: −50.39 to 121.00), showing no mediation. Adjusted (Table S2, Supplemental Digital Content, https://links.lww.com/MD/P950) and restricted (Table S3, Supplemental Digital Content, https://links.lww.com/MD/P950) results were similar (NIE: CNY 36.63 and CNY 21.15, respectively), reflecting low emergency room utilization.

For the number of CD-related inpatient stays, the unadjusted TE was − 1.70 (95% CI: −2.14 to − 1.25), with an NIE of − 0.85 (95% CI: −1.33 to − 0.37) and NDE of − 0.85 (95% CI: −1.46 to − 0.23), with a proportion mediated of 49.99% (95% CI: 18.8%–81.2%). Adjusted results (Table S2, Supplemental Digital Content, https://links.lww.com/MD/P950) showed a similar NIE (−0.84; 95% CI: −1.32 to − 0.36), and the restricted analysis (Table S3, Supplemental Digital Content, https://links.lww.com/MD/P950) showed a stronger NIE (−1.31; 95% CI: −1.99 to − 0.62), with a proportion mediated of 77.33%, indicating partial mediation.

For the number of CD-related outpatient visits, the unadjusted TE was an increase of 6.31 visits (95% CI: 5.25 to 7.36), with an NIE of 1.05 (95% CI: −0.14 to 2.23; *P* = .084) and NDE of 5.26 (95% CI: 3.69 to 6.83), with a proportion mediated of 16.61% (*P* = .09). Adjusted analysis (Table S2, Supplemental Digital Content, https://links.lww.com/MD/P950) showed a similar NIE (1.09; 95% CI: −0.06 to 2.24; *P* = .067), and the restricted analysis (Table S3, Supplemental Digital Content, https://links.lww.com/MD/P950) showed a significant NIE (2.90; 95% CI: 1.19 to 4.61; *P* < .001), with a proportion mediated of 47.03%.

For the number of CD-related emergency room visits, the unadjusted TE was not significant (0.02; 95% CI: −0.20 to 0.25), with nonsignificant NDE (−0.14; 95% CI: −0.47 to 0.20) and NIE (0.16; 95% CI: −0.09 to 0.42). Adjusted (Table S2) and restricted (Table S3) results were similar (NIE: 0.16 and 0.14, respectively), showing no mediation.

For CD-related inpatient length of stay, the unadjusted TE was − 2.25 days (95% CI: −3.74 to − 0.76), with an NIE of − 1.18 (95% CI: −2.85 to 0.50; *P* = .167) and a nonsignificant NDE (−1.07; 95% CI: −3.30 to 1.15), with a proportion mediated of 52.28%. Adjusted results (Table S2, Supplemental Digital Content, https://links.lww.com/MD/P950) showed a similar NIE (−1.14; 95% CI: −2.82 to 0.54), and the restricted analysis (Table S3, Supplemental Digital Content, https://links.lww.com/MD/P950) showed a nonsignificant NIE (−1.28; 95% CI: −3.61 to 1.05), indicating partial mediation.

For UST persistence, the unadjusted TE showed a reduced hazard of discontinuation (hazard ratio [HR]: 0.48; 95% CI: 0.36 to 0.63), with a significant NDE (HR: 0.37; 95% CI: 0.366 to 0.38) but nonsignificant NIE (HR: 1.28; 95% CI: 0.99 to 1.66; *P* = .064), indicating no mediation. Adjusted (Table S2, Supplemental Digital Content, https://links.lww.com/MD/P950) and restricted (Table S3, Supplemental Digital Content, https://links.lww.com/MD/P950) analyses showed similar nonsignificant NIEs (HR: 0.25 and 0.27, respectively), suggesting that improved persistence was driven by factors other than treatment setting, such as access to care.

The exposed group was more likely to receive all CD treatment in outpatient settings (adjusted odds ratio: 61.18; 95% CI: 16.96–220.65). Outpatient treatment was associated with lower hospitalization costs (adjusted mean difference: −CNY 13,839; 95% CI: −21,077 to − 6602) and fewer inpatient stays (adjusted mean difference: −1.50; 95% CI: −2.11 to − 0.89]). Sensitivity analyses adjusting for gender and surgery history (Table S2, Supplemental Digital Content, https://links.lww.com/MD/P950) and restricting to single-setting treatment (Table S3, Supplemental Digital Content, https://links.lww.com/MD/P950) confirmed the robustness of these findings.

## 4. Discussion

This cohort study aimed to evaluate whether a balanced insurance policy (Hangzhou Medical Insurance, offering similar coverage for inpatient and outpatient care) reduces total CD treatment costs and improves UST persistence compared to an inpatient-only group (Zhejiang Province Card Insurance, covering inpatient care but not outpatient care) among CD patients initiating UST in 2022 at Sir Run Run Shaw Hospital, Hangzhou, China, and whether these effects are mediated by a shift to outpatient treatment. Our key findings are: the balanced insurance group had lower CD-related hospitalization costs, higher CD-related outpatient costs, fewer inpatient stays and more outpatient visits, and a 72.8% lower risk of UST discontinuation compared to the inpatient-only group. First, the balanced insurance group incurred significantly lower CD-related hospitalization costs (mean difference: −CNY 6015.04; 95% CI: −11,189.46 to − 840.62) compared to the inpatient-only group. This aligns with prior studies showing that insurance policies favoring outpatient care reduce hospitalization costs for IBD.^[13,14]^ For instance, Park et al.^[14]^ found that outpatient-focused insurance reduced IBD-related inpatient costs by shifting care to less resource-intensive settings. Our findings are consistent due to China’s 2021 insurance reforms promoting outpatient coverage,^[12]^ which likely encouraged outpatient UST administration in the balanced group. Unlike prior studies, we used mediation analysis to confirm that this cost reduction was fully mediated by increased outpatient treatment (NIE: −CNY 10,328.11; 95% CI: −16,129.53 to − 4526.68; Table 3), a novel approach that quantifies the role of treatment setting in cost savings.

Second, the balanced insurance group had higher CD-related outpatient costs (mean difference: CNY 220.16; 95% CI: 40.92 to 399.40) than the inpatient-only group. This is consistent with Quiros et al.,^[13]^ who reported increased outpatient costs in pediatric CD patients with insurance covering outpatient biologics. The higher costs in our study reflect increased outpatient visits for UST administration, facilitated by balanced coverage (76%–92% reimbursement for outpatient care) compared to no outpatient coverage in the control group.^[19]^ Differences may arise because Quiros et al focused on pediatric populations, whereas our study included adults with a median follow-up of 255 days. Our mediation analysis showed that 76.18% of this cost increase was mediated by outpatient treatment (Table 3), providing novel evidence that balanced insurance drives outpatient care utilization.

Third, the balanced insurance group had fewer inpatient stays (mean difference: −1.70; 95% CI: −2.14 to − 1.25) and more outpatient visits (mean difference: 6.31; 95% CI: 5.25 to 7.36) compared to the inpatient-only group. This aligns with Freeman et al.,^[16]^ who found that insurance coverage influences healthcare utilization patterns in chronic diseases. The shift to outpatient care in our study is likely due to Hangzhou balanced reimbursement (82%–92% for inpatient, 76%–92% for outpatient) reducing financial barriers to outpatient UST administration.^[19]^ Unlike Freeman et al., which examined general chronic conditions, our study focused on CD and used a restricted analysis (Table S1, Supplemental Digital Content, https://links.lww.com/MD/P950) to confirm robustness in patients receiving all treatment in 1 setting (n = 98). This study’s novelty lies in demonstrating that balanced insurance reduces inpatient stays by 49.99% through outpatient treatment mediation (Table 3). Fourth, the balanced insurance group had a 72.8% lower risk of UST discontinuation (adjusted HR: 0.27; 95% CI: 0.10–0.74) compared to the inpatient-only group, as shown in Kaplan–Meier curves (Fig. 2). This is consistent with Ventress et al.,^[9]^ who reported improved persistence with subcutaneous biologics due to easier access. However, unlike Ventress et al., which focused on vedolizumab, our study examined UST, a newer biologic in China’s National Reimbursement Drug List (2022).^[21]^ Mediation analysis revealed that this improved persistence was not significantly mediated by outpatient treatment, suggesting other factors, such as proximity to the IBD Center for Hangzhou residents, may contribute. This study uniquely identifies nontreatment-setting factors driving biologic persistence in a Chinese cohort.

## 5. Strengths and limitations

Strengths include the comprehensive IBD registry at Sir Run Run Shaw Hospital, with detailed patient data extracted from the hospital’s information system, ensuring accuracy.^[18]^ The natural experiment design minimized confounding by comparing similar patient groups. Unlike prior studies focusing only on costs,^[7]^ we reported both costs and healthcare service use, clarifying that cost savings stemmed from reduced inpatient stays. Limitations include the inability to separate medication (e.g., UST) and service costs, which may overestimate cost differences between groups, as UST costs are high but consistent across settings. However, this does not alter our conclusions, as the observed differences in hospitalization and outpatient costs are primarily driven by utilization patterns (e.g., fewer inpatient stays, more outpatient visits), which are robust to cost composition. Although the GDPs of Hangzhou and Zhejiang Province are comparable,^[12]^ residual confounding (e.g., unmeasured socioeconomic factors^[14,22,23]^ or care access at other sites) may exist.^[14]^ We mitigated this through conservative estimates, adjusting for baseline gender and gastrointestinal surgery history in all analyses and conducting sensitivity analyses (e.g., restricted analysis in Table S1, Supplemental Digital Content, https://links.lww.com/MD/P950), which yielded consistent results, reducing the impact of unmeasured confounders, though some bias may persist. The small sample size (n = 133), short follow-up (~8.5 months), and single-center design limit generalizability, reflecting UST’s recent reimbursement.^[21]^ Clinical data reliability depends on accurate coding, which may introduce errors.^[13,24,25]^

## 6. Conclusion

This study provides novel insights into the impact of balanced insurance on CD care in China. First, by quantifying the role of outpatient treatment in reducing hospitalization costs through mediation analysis, it advances understanding of cost-saving mechanisms beyond prior descriptive studies. Second, it demonstrates that balanced insurance drives outpatient utilization, offering evidence specific to China’s healthcare context. Third, it provides actionable policy insights by showing that outpatient treatment reduces inpatient stays, informing efficient resource allocation. Finally, it uniquely identifies nontreatment-setting factors, such as proximity or care coordination, as drivers of UST persistence, filling a gap in the literature on biologic adherence in Chinese CD patients. Policymakers should equalize inpatient and outpatient coverage to optimize CD care efficiency. Future multi-center studies with longer follow-up should include patients with UC, as UC and CD are both IBDs that share biologic treatments like UST and face similar insurance-related access challenges, enhancing generalizability. These studies should also explore other biologics and outcomes like surgery or quality of life to guide China’s insurance reforms.

## Author contributions

**Conceptualization:** Yue Gao.

**Data curation:** Jihao Shi, Yongjia Zhuo, Qian Cao.

**Formal analysis:** Yue Gao.

**Funding acquisition:** Jihao Shi, Qian Cao.

**Methodology:** Yue Gao.

**Project administration:** Jihao Shi, Kangchen Lv.

**Resources:** Yipeng Pan, Qian Cao.

**Supervision:** Xiaohan Hu.

**Validation:** Xiaohan Hu.

**Writing – original draft:** Yue Gao.

**Writing – review & editing:** Xiaohan Hu.

## Supplementary Material


