# Supplementary material for: Balanced inpatient and outpatient reimbursement versus hospitalization-favored insurance reduces Crohn disease costs and improves biologic drug persistence: A mediation analysis
Source: Medicine (Baltimore). 2025 Sep 12;104(37):e44475. doi: 10.1097/MD.0000000000044475 (PMC12440511; doi:10.1097/MD.0000000000044475)

**Supplementary Figure S1. Directed Acyclic Graph of Mediation Analysis for Outpatient Treatment Effects on Outcomes in Crohn’s Disease Patients Initiating Ustekinumab by Insurance Type at Sir Run Run Shaw Hospital, Hangzhou, China, 2022**


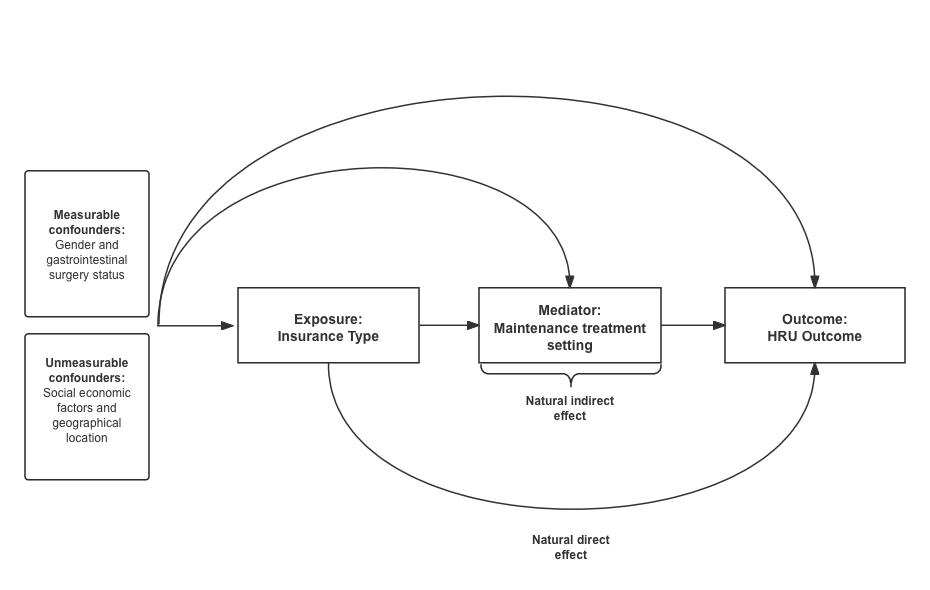

Supplement: Supplementary file 1 [file medi-104-e44475-s001.docx]
