# Supplementary material for: Balanced inpatient and outpatient reimbursement versus hospitalization-favored insurance reduces Crohn disease costs and improves biologic drug persistence: A mediation analysis
Source: Medicine (Baltimore). 2025 Sep 12;104(37):e44475. doi: 10.1097/MD.0000000000044475 (PMC12440511; doi:10.1097/MD.0000000000044475)
Supplement: Supplementary file 2 [file medi-104-e44475-s002.docx]

**Table S1. Healthcare Expenditure and Resource Use Outcomes for Crohn’s Disease Patients Initiating Ustekinumab Receiving All Treatment in One Setting (Inpatient or Outpatient) at Sir Run Run Shaw Hospital, Hangzhou, China, 2022**

|  | **Cost and health resource use (HRU), mean (95% CI)** | | | | | |
| --- | --- | --- | --- | --- | --- | --- |
|  | **Total included patients (n=98)** | **Balanced Insurance Group (n=48)** | **Inpatient-Only Group (n=50)** | **Cost/HRU difference^b^** | **Adjusted cost/HRU difference^d^** | |
| Healthcare expenditure outcome, CNY^a^ |  |  |  |  |  |  |
| Total CD treatment cost | 13,202.90 (10,601.45, 15,804.35) | 10,111.90 (7,128.80, 13,095.01) | 15,872.40 (11,801.92, 19,942.88) | -5,760.50 (-10,895.71, -625.28)^c^ | -6,348.94 (-11,658.78, -1,039.10)^c^ | |
| CD-related hospitalization cost | 12,788.10 (10,180.32, 15,395.89) | 9,571.78 (6,551.00, 12,592.57) | 15,565.84 (11,511.08, 19,620.59) | -5,994.05 (-11,133.65, -854.46)^c^ | -6,603.81 (-11,917.56, -1,290.06)^c^ | |
| CD-related outpatient cost | 347.25 (250.16, 444.33) | 466.13 (312.50, 619.76) | 244.58 (123.32, 365.83) | 221.55 (30.16, 412.95) ^c^ | 232.55 (33.80, 431.31)^c^ | |
| CD-related emergency room cost | 67.55 (26.69, 108.41) | 73.99 (21.10, 126.89) | 61.99 (-0.22, 124.20) | 12.00 (-70.25, 94.25) | 22.31 (-62.91, 107.54) | |
| Healthcare resource use outcome |  |  |  |  |  |  |
| Number of inpatient stays | 3.41 (3.14, 3.69) | 2.51 (2.26, 2.76) | 4.20 (3.82, 4.57) | -1.69 (-2.15, -1.23)^c^ | -1.61 (-2.09, -1.14)^c^ | |
| CD-related outpatient visit number | 5.09 (4.31, 5.87) | 8.40 (7.36, 9.45) | 2.23 (1.72, 2.73) | 6.18 (5.08, 7.27)^c^ | 6.51 (5.41, 7.61)^c^ | |
| CD-related emergency room visit number | 0.24 (0.11, 0.36) | 0.26 (0.09, 0.44) | 0.21 (0.04, 0.38) | 0.05 (-0.19, 0.29) | 0.07 (-0.18, 0.33) | |
| CD-related inpatient length of stay | 6.36 (5.60, 7.11) | 5.09 (4.10, 6.08) | 7.45 (6.38, 8.53) | -2.37 (-3.83, -0.90)^c^ | -2.15 (-3.66, -0.63)^c^ | |

**Footnotes**:
a. CNY: Chinese Yuan; CI: Confidence Interval. Numbers in brackets represent 95% confidence intervals unless otherwise specified.
b. The restricted analysis includes patients receiving all ustekinumab (UST) treatment in one setting (inpatient or outpatient) during follow-up in 2022.
c. Statistically significant difference at P < 0.05.
d. Adjusted for baseline gender and gastrointestinal surgery history. The balanced insurance group refers to Hangzhou Medical Insurance (similar coverage for inpatient and outpatient care); the inpatient-only group refers to Zhejiang Province Card Insurance (covers inpatient care but not outpatient care).

**Table S2. Adjusted Mediation Analysis of Outpatient Treatment Effects on Outcomes for Crohn’s Disease Patients Initiating Ustekinumab by Insurance Type at Sir Run Run Shaw Hospital, Hangzhou, China, 2022**

| **Treatment setting's mediating effect on different outcomes ^a,b^** | **Mean difference (95% CI)** | | | **Proportion mediated**^e^ **(%)** |
| --- | --- | --- | --- | --- |
|  | **Total effect** | **Natural direct effect** | **Natural indirect effect** |  |
| Healthcare expenditure outcome |  |  |  |  |
| CD-related treatment cost | -6,406.10 (-12,950.68, 138.48) | 3,813.13 (-3,648.89, 11,275.14) | -10,219.22 (-16,043.37, -4,395.08)^d^ | NA |
| CD-related hospitalization cost | -6,647.08 (-13,242.90, -51.27)^d^ | 3,778.06 (-3,678.15, 11,234.26) | -10,425.14 (-16,255.61, -4,594.67)^d^ | NA |
| CD-related outpatient cost | 227.69 (32.56, 422.83)^d^ | 58.40 (-212.50, 329.30) | 169.29 (-34.15, 372.73) | 74.35 |
| CD-related emergency room cost | 13.29 (-66.02, 92.60) | -23.33 (-138.93, 92.27) | 36.63 (-49.49, 122.74) | NA |
| Healthcare resource use outcome |  |  |  |  |
| CD-related hospitalization number | -1.64 (-2.19, -1.09)^d^ | -0.80 (-1.42, -0.18) | -0.84 (-1.32, -0.36)^d^ | 51.27^d^ |
| CD-related outpatient visit number | 6.53 (5.41, 7.65)^d^ | 5.44 (3.92, 6.97)^d^ | 1.09 (-0.06, 2.24) | 16.68^c^ |
| CD-related emergency room visit number | 0.04 (-0.20, 0.28) | -0.12 (-0.46, 0.22) | 0.16 (-0.09, 0.42) | NA |
| CD-related inpatient length of stay | -2.02 (-3.61, -0.44)^d^ | -0.88 (-3.12, 1.36) | -1.14 (-2.82, 0.54) | 56.42 |
| Persistence | -0.73 (-1.02, -0.45)^d^ | -0.98 (-1.01, -0.96)^d^ | 0.25 (-0.02, 0.52) | NA |

a. The exposed group is compared with the control group

b. The mediating effect of receiving all treatment under outpatient setting during follow-up is compared with receiving at least one treatment under inpatient setting (reference group)

c. P value: 0.067

d. Statistically significant effect at a P value of less than 0.05

e. Proportion mediated: the measure is problematic when the natural direct effect and natural indirect effect operate in different directions. One can then obtain a proportion mediated much larger than 100%, and the measure is no longer really meaningful.

**Table S3. Mediation Analysis of Outpatient Treatment Effects on Outcomes for Crohn’s Disease Patients Initiating Ustekinumab Receiving All Treatment in One Setting (Inpatient or Outpatient) at Sir Run Run Shaw Hospital, Hangzhou, China, 2022**

| **Treatment setting's mediating effect on different outcomes ^a,b^** | **Mean difference (95% CI)** | | | **Proportion mediated**^e^ **(%)** |
| --- | --- | --- | --- | --- |
|  | **Total effect** | **Natural direct effect** | **Natural indirect effect** |  |
| Healthcare expenditure outcome |  |  |  |  |
| CD-related treatment cost | -5,760.50 (-10,925.54, -595.45)^d^ | 10,814.76 (1,827.16, 19,802.36)^d^ | -16,575.26 (-24,500.37, -24,500.37)^d^ | NA |
| CD-related hospitalization cost | -5,994.05 (-11,165.03, -823.08)^d^ | 10,894.75 (1,921.44, 19,868.06)^d^ | -16,888.80 (-24,812.81, -24,812.81)^d^ | NA |
| CD-related outpatient cost | 221.55 (30.15, 412.96)^d^ | -70.84 (-426.63, 284.96) | 292.39 (-11.36, -11.36)^c^ | NA |
| CD-related emergency room cost | 12.00 (-70.10, 94.11) | -9.15 (-163.96, 145.66) | 21.15 (-110.11, -110.11) | NA |
| Healthcare resource use outcome |  |  |  |  |
| CD-related hospitalization number | -1.69 (-2.15, -1.23)^d^ | -0.38 (-1.16, 0.40) | -1.31 (-1.99, -1.99)^d^ | 77.33^d^ |
| CD-related outpatient visit number | 6.18 (5.07, 7.28)^d^ | 3.27 (1.29, 5.26)^d^ | 2.90 (1.19, 1.19)^d^ | 47.03^d^ |
| CD-related emergency room visit number | 0.05 (-0.19, 0.29) | -0.09 (-0.55, 0.37) | 0.14 (-0.24, -0.24) | NA |
| CD-related inpatient length of stay | -2.37 (-3.83, -0.91)^d^ | -1.08 (-3.82, 1.65) | -1.28 (-3.61, -3.61) | 54.23 |
| Persistence | -0.71 (-1.01, -0.42)^d^ | -0.98 (-1.01, -0.96)^d^ | 0.27 (-0.01, -0.01) | NA |

a. The exposed group is compared with the control group

b. The mediating effect of receiving all treatment under outpatient setting during follow-up is compared with receiving at least one treatment under inpatient setting (reference group).

c. P value: 0.059.

d. Statistically significant effect at a P value of less than 0.05.

e. Proportion mediated: the measure is problematic when the natural direct effect and natural indirect effect operate in different directions. One can then obtain a proportion mediated much larger than 100%, and the measure is no longer really meaningful.
